# A Comparison of Three Executive Function Batteries in a Preschool-Aged Sample

**DOI:** 10.3390/children11070811

**Published:** 2024-07-02

**Authors:** Laura J. Kuhn, Marie Camerota, Michael T. Willoughby, Clancy Blair

**Affiliations:** 1 FPG Child Development Institute, University of North Carolina at Chapel Hill, Chapel Hill, NC 27599, USA; 2 Center for Developmental Science, University of North Carolina at Chapel Hill, Chapel Hill, NC 27599, USA; marie_camerota@brown.edu; 3 RTI International, Research Triangle Park, NC 27709, USA; mwilloughby@rti.org; 4 Department of Applied Psychology, New York University, New York, NY 10012, USA

**Keywords:** executive function, preschool, cognitive assessment

## Abstract

There is great interest in the development of executive function (EF) in the preschool period. Accordingly, multiple performance-based measures of EF have been developed for this age group, yet little is known about how they compare to one another. This study used a large and diverse sample of 3-to-5-year-old children (*N* = 846), who completed subtests of the National Institutes of Health’s Toolbox Cognition Battery (NTCB), the Wechsler Preschool and Primary Scale of Intelligence (WPPSI-IV), and the EF Touch battery. Scores across the three batteries were compared and associations with age, income, and race/ethnicity were examined. Results revealed that (1) the three tasks were moderately correlated (*r* = 0.44–0.51, all *p* < 0.001), but children had higher mean accuracy scores on EF Touch than on the NTCB or the WPPSI-IV. (2) Mean accuracy scores on all batteries were linearly associated with child age (all *F* > 32.68, all *p* < 0.0001). (3) Comparisons by income and race/ethnicity showed lower accuracy for low-income children on the WPPSI-IV and lower accuracy for White children on the NTCB. Across all batteries, there was consistently lower accuracy for Hispanic children. In conclusion, the three batteries we examined performed similarly across several metrics. EF Touch may be more appropriate for younger children, while the NTCB performed well with older children.

## 1. Introduction

There is great interest in the development of executive functions (EFs) during the preschool period [1,2,3]. EFs refer to a set of abilities that typically includes working memory, attention shifting, and inhibitory control in the service of problem-solving and promoting self-regulatory skills [4]. The preschool period is noteworthy due to the rapid development and partitioning of executive abilities during this time [5,6]. Several studies have documented that EF begins as a unified construct [7,8] which begins to differentiate around age 5 [9]. EF has a protracted course of development with skills maturing well into adolescence [10].

Preschool EFs are related to several aspects of school success, including academic achievement, prosocial abilities, and positive emotional outcomes [11]. Consequently, there has been a proliferation of EF measures that are available for use with younger children. In the mid-1990s, there was a new-found interest in the creation of EF measures for preschoolers [12]. This interest continued through the 2000s with an increase in both the developmental theory supporting the emergence of EF in young children [6,13] and the development of measures that could document these developmental changes [14]. More recently, the focus has been on measuring EF at even younger ages [15,16]. As various EF measures continue to be developed and validated, it is unclear how they are related to one another and whether they exhibit similar psychometric properties. With increasing interest in the assessment of EF across many fields (e.g., psychology, pediatrics, education), researchers and clinicians could benefit from additional information to determine whether existing EF batteries are appropriate for specific populations. Our research team has developed the EF Touch battery [17] for use with preschool-aged children. The goal of this manuscript is to compare EF Touch to alternative batteries and to provide percentile scores for future EF Touch users.

### 1.1. EF Touch

Though relatively recent in its inception, the EF Touch battery has already been used over a dozen times [2,18,19,20]. EF Touch is a computerized battery of seven EF tasks tapping aspects of inhibitory control, attention shifting, and working memory (for full description see Willoughby et al., 2010) [17]. Several studies have evaluated aspects of EF Touch for potential users. It has demonstrated evidence of strong psychometric properties, including adequate test–retest reliability [21] and predictive validity [22]. The battery was explicitly developed to be used with low-income populations [17] and has been used with preschoolers with parent-reported disabilities [23]. EF Touch has also demonstrated the ability to detect developmental changes from the ages of three to five years [2,24]. Finally, relevant research has identified numerous early predictors of performance on the battery, including poverty, parenting, salivary cortisol, and child and parent language [25,26,27,28].

The EF Touch battery is free to use, appropriate for ages from 3 to 5 years, has been widely shared with others within the US and internationally, and has been translated into several languages, such as Spanish and Chinese [29]. Despite this popularity, research has yet to examine how child performance on the EF Touch battery compares to performance on other widely used EF batteries.

### 1.2. Comparison with Alternative EF Batteries

Two alternative batteries include the National Institutes of Health’s Toolbox Early Childhood Cognition Battery (NT-ECCB) and the Wechsler Preschool and Primary Scale of Intelligence 4th edition (WPPSI-IV). The NT-ECCB is a computerized assessment including two measures of EFs: the Dimensional Change Card Sort (DCCS) and the Flanker task. The NT-ECCB is validated for ages from 3 to 85 years and has demonstrated adequate test–retest reliability, as well as convergent and discriminant validity [30], yet there is some concern about floor effects for the EF subtests when administered to the youngest children in the validated range [31]. The NT-ECCB has been used with children with traumatic brain injuries [32] and disabilities [33]. Additionally, norm reference scores are available for a variety of subpopulations [34]. The battery has also been translated into Spanish [35]. The NT-ECCB is a relevant battery for comparison with the EF Touch battery because of its popularity, validation with a wide age range, and utilization of two well-known tasks [36,37].

Another alternative to EF Touch is the WPPSI-IV, which includes four subtests that provide measures of working memory and fluid reasoning. Although not interchangeable with EF, the construct of fluid reasoning shares considerable conceptual overlap and common loadings in factor analysis [38,39]. The WPPSI-IV has been validated for use with children aged 2 years and 6 months through 7 years and 7 months [40]. The tests have demonstrated adequate test–retest reliability and validity, and normative reference scores are available for a variety of subpopulations [40]. The WPPSI-IV is another important battery for comparison with the EF Touch battery because it represents one of the few batteries that have been developed, evaluated, and normed by professional test developers. This comparison helps us understand how EF Touch, which was developed by substantive researchers, compares to a normative, rigorously evaluated instrument that is often used by practitioners.

Despite similarly strong psychometric properties, these three batteries differ widely in terms of cost, materials, scoring, and administration burden. As mentioned previously, EF Touch software is free, although it does require the use of a touchscreen laptop and an auxiliary monitor for administration [29]. In contrast, the NT-ECCB is available as an application, with a subscription fee of $599.99 for use on two iPads with increasing fees up to $2499.99 for use on 10 iPads [41]. The WPPSI-IV is similarly proprietary, as it requires specific testing materials (e.g., booklets, stimuli) that are available for purchase as a kit. The price of a single testing kit is $1638.70. An application for web-based administration of WPPSI-IV is available for purchase but also requires the purchase of stimuli testing materials for some subtests (including those assessing fluid reasoning). Pricing for the application varies based on the number of subtests used and sample size [42]. The NT-ECCB and EF Touch require minimal administrator training, as an electronic script is provided and scores are automatically generated. Alternatively, the WPPSI-IV requires more effort for administration because of the use of manipulatives and the requirement that the experimenter establish a basal level and apply discontinue criteria to generate scores.

### 1.3. Current Study

Given the multitude of EF tasks available to researchers and practitioners, a direct comparison among measures that vary in price and administration demands is needed. The current study explicitly compared children’s performance on the EF Touch battery with that of the more commonly used NT-ECCB and WPPSI-IV. Comparisons included practical issues that may impact administration and interpretation. For example, researchers and practitioners are often concerned with psychometric issues such as the presence of floor or ceiling effects, and whether tasks can be used within specific age ranges. We compared the free EF Touch battery to the proprietary batteries (NT-ECCB, WPPSI-IV) using several metrics, including mean task and battery scores, task completion rates, and floor and ceiling effects. We then compared these same parameters in relevant subsamples (males vs. females, White vs. minoritized children, 3- vs. 4- vs. 5-year-olds, children from low- vs. not-low-income households) to understand how the batteries perform among diverse subpopulations. No study to date has conducted a direct comparison of this nature, using the same batteries within the same sample, though this information is crucial for researchers and practitioners to weigh the advantages and disadvantages of various performance-based measures. As a secondary objective, we aimed to provide percentile scores for the EF Touch tasks and overall battery to facilitate its use among researchers and clinicians.

## 2. Methods

### 2.1. Participants and Procedures

This study used a quota-based sampling approach to recruit children (*N* = 846 children) from preschools in New York and North Carolina. Using data from the 2012 US Census, we established a distribution of children (from 0 to 5 years) with respect to household income (i.e., low income, ≤100%; near-low income, 100–200%; and not-low income, ≥200% of the US federal poverty threshold for a given household size), race (i.e., White, African American/Black, Asian, Native American, Pacific Islander), and ethnicity (i.e., Hispanic, non-Hispanic). We then used this information to generate 180 mutually exclusive cells (3 income × 5 race × 2 ethnicity × 3 age × 2 gender) that served to guide child enrollment. Consistent with a quota sampling approach, the intent was not to recruit exact numbers of children into any given cell (in fact, all eligible and interested children were enrolled). Rather, the intent was to recruit a large convenience sample of children who exhibited diversity with respect to race, ethnicity, household income, age, and gender using the expected cell counts as targets. In general, we met or exceeded the target number of children for most race × ethnicity × income level cells, with the exception that White children at all income levels were under-recruited regarding their target cell counts. The final sample of 846 children varied with respect to age (36% 3-, 45% 4-, and 19% 5-year-olds), gender (50% male), race (60% White, 31% African American, 7% Asian, 1% Native American, 1% Pacific Islander), ethnicity (20% Hispanic, 80% non-Hispanic), and household income level (25% low income, 21% near-low income, 54% not-low income). Within age groups, there were more male and non-White children in the 5-year-old group (58% male; 51% White), compared to the 3-year-old (46% male; 63% White) and 4-year-old (50% male; 63% white) groups. Chi-square tests between the 4- and 5-year-old age groups revealed these differences to be significant (χ^2^ (2) ≥ 6.40, all *p* < 0.05).

Rolling recruitment occurred during the 2014 and 2015 calendar years by approaching the directors of center-based preschools that served children 3–5 years of age from a variety of communities. Center directors distributed consent forms to parents of children in the target age range. Interested parents returned their consent forms to the preschools and were contacted via telephone by research staff for a 10-to-15-minute screening conversation. During this screening, demographic information was provided by parents about themselves and their child, and a questionnaire was completed. Children who were outside of the target age (i.e., 3.0–5.9 years of age), who had physical or mental disabilities that prohibited their ability to participate in direct assessments, or who did not speak English were not eligible to participate. Teachers and parents determined child participation eligibility based on whether the child could touch a computer screen in response to a prompt.

Following the recruitment phone call, children participated in a one-time assessment of EF abilities in their preschool. Of the 924 families who completed recruitment phone calls and were eligible to participate, 92% (*N* = 846) were tested. Due to the large number of planned assessments, it was infeasible to assign each child to complete all tasks because of anticipated fatigue effects. This practical issue was addressed in two ways. First, older children were administered more tasks than younger children to reduce testing time (e.g., 3-year-olds were not administered NT-ECCB). Second, we utilized a planned missing design where children were randomized to complete a subset of EF tasks (e.g., NT-ECCB, WPPSI-IV). The order in which tasks were administered was counterbalanced. As a result, there were no differences in gender, income status, race, or ethnicity when comparing children who did versus did not complete each task battery (all *p* > 0.05). Before child assessments began, written consent was obtained from all parents/guardians of the participants. At the time of assessment, assent was obtained from child participants before participation in the study and children could decline to participate. Testing sessions were completed by a research assistant and took approximately 30–45 min for 3-year-old and 45–60 min for 4- and 5-year-old children. Children were assessed in a quiet area with a research assistant, often outside of the classroom (i.e., hallway or empty room). For each completed consent form, preschool centers received $5, parents received $40 for the completion of the screening phone call, and children received a small gift for their participation.

### 2.2. Measures

#### 2.2.1. Executive Function Touch (EF Touch)

EF Touch is a computerized battery of EF tasks that were initially created, administered, and extensively studied in paper and pencil (i.e., “flip book”) formats [17]. The computerization of tasks improved the efficiency (i.e., a single data collector with minimal training can administer tasks; touchscreen monitors eliminate data keying; data are available in comma delimited format), standardization (i.e., task instructions, examiner prompts, and the administration of training items and resulting decisions about whether a task should be completed are all standardized), and sensitivity (i.e., more difficult items were added to some tasks; item-level reaction time data were recorded with an interest in differentiating ability level) of the previous battery of tasks [22]. Six of the seven tasks were used in the current study.

The EF Touch program requires a capacitive touchscreen monitor to record child responses and a standard monitor to display the administration script read by the assessor (see Willoughby et al., 2017 for detailed information) [21].

The EF Touch battery is modular in nature (i.e., any number of tasks can be administered in any desired order), and each EF task takes 3–7 min to complete. Two warm-up tasks (1–2 min/each) are typically administered first to acclimate children to using the touch screen. In addition, each task required the child to successfully complete a series of practice trials before proceeding to test items to ensure the child understood the task instructions. Children were given two chances to repeat the practice trials for each task. Because each of the tasks in the EF Touch program has been described and extensively studied in detail elsewhere, only abbreviated descriptions are provided here. Unless noted, the total mean accuracy of all items was used to index a child’s performance (see Willoughby et al., 2017 for a detailed description of tasks and scoring) [21].

##### Spatial Conflict Arrows

This 36-item spatial conflict task measured inhibitory control. Two buttons appeared on the left- and right-most sides of the touchscreen monitor. Children were instructed to touch the button to which the task stimulus (i.e., an arrow) was pointing. Arrows either appeared above the button to which they were pointing (congruent condition), the opposite button (incongruent condition), or in mixed locations. Mean accuracy for the incongruent items indexed the performance.

##### Silly Sounds Stroop

This 17-item Stroop-like task measured inhibitory control. For each item, a picture of a dog and a picture of a cat was displayed (left and right placement varied across trials) with the sound of either a barking dog or a meowing cat. Children were instructed to touch the animal that did not make the sound (e.g., touch the cat when hearing a dog bark).

##### Animal Go/No-Go

This 40-item go/no-go task measured inhibitory control. Pictures of animals were displayed, and children were asked to touch a centrally located button every time they saw an animal (the “go” response), except when the animal was a pig (the “no-go” response). Mean accuracy across all no-go trials indexed task performance.

##### Working Memory Span

This 18-item span task measured working memory. Each item contained a picture of two or more houses which contained a picture of an animal and a colored dot, or a colored animal. Children were asked to label the animal and color in each house. After a brief delay, the houses were displayed again without any content and the child was asked to recall either the animal or color that was in each house (i.e., the non-recalled contents served as a distraction). The number of houses increased from 2-, 3-, 4-, to 6-house trails.

##### Pick the Picture

This 32-item self-ordered pointing task measured working memory. Children were presented with picture sets that varied in length from 2, 3, 4 to 6 pictures per set. For each item, children were instructed to first touch any picture and then on subsequent trials (within that set) to pick a picture that had not yet been touched.

##### Something’s the Same

This 30-item task measured attention shifting and flexible thinking. Children were presented with two pictures similar in respect to their color, shape, or size. Then, a third picture was presented alongside the original two pictures, and the child was asked to select which of the two original pictures was similar to the new picture, thus matching along one of the other dimensions (e.g., color, shape, or size). In the last 10 items, the three pictures were presented all at once.

#### 2.2.2. NIH Toolbox Early Childhood Cognition Battery (NT-ECCB)

The NT-ECCB is a computerized assessment comprised of seven tasks that measure eight abilities within six major cognitive domains. Two of these tasks measure EF. Because detailed information on the development of these tasks exists elsewhere [43], here we offer a summary pertaining to task administration. In the current study, due to time constraints, the NT-ECCB was only administered to 4- and 5-year-olds.

##### Dimensional Change Card Sort Test (DCCS)

The DCCS measures cognitive flexibility by asking children to sort pictorial stimuli by multiple, changing dimensions. Children were presented with a central stimulus (e.g., a yellow rabbit) on a laptop computer and asked to match it to one of two lateralized target stimuli based on either color (e.g., a yellow truck) or shape (e.g., a blue rabbit). The sorting rule was presented both visually and auditorily (via computerized recording). Children indicated their responses by pressing one of two keyboard buttons that were spatially congruent with the target stimuli.

In a series of practice trials, children first demonstrated the ability to sort stimuli by one dimension (e.g., color) while receiving feedback on their performance. Children were required to identify three out of four practice items correctly for the first dimension. The practice was repeated up to three times. Once children met this criterion, they received a second practice block for the other sorting dimension (e.g., shape). Once again, children were required to identify three out of four practice items correctly, and the practice block could be repeated up to three times. Once children passed the two practice phases, they received five pre-switch items, which were similar in structure to the second practice block (e.g., children were required to sort new pictures based on shape). Children received no feedback on their performance. If children responded correctly to four of five pre-switch items, they proceeded to the post-switch block, in which they were required to sort by the other dimension (in this example, color). If children successfully completed four of five post-switch items, they proceeded to a mixed block. In the mixed block, children were asked to sort stimuli flexibly based on either color or shape. This block included 30 items that were presented in a pseudorandom order. Accuracy was scored for items in the pre-switch, post-switch, and mixed block (40 items total).

##### Fish Flanker

The Fish Flanker task measures inhibitory control and executive attention by requiring children to indicate the left–right orientation of a central stimulus while ignoring the orientation of either congruent or incongruent flanking stimuli. The version of the task created for the NT-ECCB includes 25 trials with fish stimuli and 25 trials with arrow stimuli. Similar to the DCCS, children were asked to indicate their response by pressing one of two keyboard buttons that were spatially congruent with their answer (i.e., on the left or right side of the keyboard).

The Fish Flanker task included a practice block of four items (two congruent and two incongruent). Children had to respond correctly to at least 3 of the 4 practice items to proceed to the test items. The practice was repeated up to three times. Children who met the criterion then received 25 fish trials (16 congruent and 9 incongruent) presented in a pseudorandom order. Children who answered at least 5 of the 9 incongruent fish trials correctly proceeded to the arrow trials. The structure of the arrow trials was the same as the fish trials. Because of demonstrated fatigue effects [43], only the first 20 items in the fish and arrow blocks were scored (40 items in total).

#### 2.2.3. Weschler Preschool and Primary Scale of Intelligence 4th Edition (WPPSI-IV)

The WPPSI-IV [40] comprises 15 tasks which assess 3 core abilities before 4 years of age and 5 core abilities after 4 years of age. It includes two tests of working memory and two tests of fluid reasoning, a construct that exhibits considerable overlap with EF [39]. Fluid reasoning tasks are only administered to children of 4 and older. The WPPSI-IV has been widely used and studied, and thus we offer only a brief description of the tasks used in the current study.

##### Zoo Locations

In the Zoo Locations task, an experimenter places cards depicting various animals in designated ‘homes’ on a mat for a specified period. Then, the experimenter hands the cards to the child and asks him or her to put the animals where they live. The task begins with 1 sample item followed by 20 test items. The child is given two trials on the sample item and the first two test items, and the experimenter can provide feedback on his/her performance. The child must complete at least one of the first two test items correctly to proceed. The task is discontinued after two consecutive incorrect responses.

##### Picture Memory

In the Picture Memory task, the experimenter shows the child one or more target pictures for a specific period and then asks the child to select the target picture(s) from a larger set of pictures. Children younger than age four are presented with one sample item followed by 35 test items. Children aged four and older are presented with one sample item and then begin at test item 7. If the child does not receive a perfect score on both test items 7 and 8, administration proceeds in reverse until the child answers two consecutive items correctly. For all age groups, the experimenter can provide feedback on test items 1, 2, 7, and 8. The task is discontinued after three consecutive incorrect responses.

##### Matrix Reasoning

In the Matrix Reasoning task, the child views an incomplete matrix of pictures and selects the picture that best completes the matrix. Children younger than age five complete 3 sample items followed by 26 test items. Children aged five years and older complete 3 sample items and then start at test item 4. If the child does not receive a perfect score on both test items 4 and 5, administration proceeds in reverse until the child answers two consecutive items correctly. For all age groups, the experimenter only provides feedback on the sample items. The task is discontinued after three incorrect responses.

##### Picture Concepts

In the Picture Concepts task, children are shown two or three rows of pictures and must select one picture from each row that forms a group. For example, the child might select a cat in one row and a dog in another row because they are both pets. There is only one correct pairing per item. The task consists of 2 sample items followed by 27 test items. The experimenter can only provide feedback on the two sample items, and the task is discontinued after three consecutive incorrect responses.

### 2.3. Task Scoring

#### 2.3.1. Accuracy

Tasks from each battery were scored in accordance with published scoring guidelines. Apart from Spatial Conflict Arrows and Animal Go/No Go, accuracy scores for tasks in the EF Touch battery represented the proportion of correct answers across all trials, resulting in scores that ranged from 0 to 1. Accuracy on the Spatial Conflict Arrows task was based on the proportion of correct answers in incongruent trials. Accuracy on the Animal Go/No Go task was based on the proportion of correct responses on no-go trials. For the NT-ECCB, accuracy scores were calculated as (0.125 × number of correct responses), yielding scores that ranged from 0 to 5. Scaled scores were calculated for each WPPSI-IV task based on the child’s age and number of correct responses, in accordance with norming guidelines. To facilitate comparisons of performance across tasks in different batteries, we also calculated proportion scores for NT-ECCB and WPPSI-IV tasks (e.g., the number of correct items divided by total items), resulting in a similar scoring metric for all 3 batteries.

#### 2.3.2. Failure to Pass Practice/Achieve Basal Level of Performance

EF Touch and NT-ECCB tasks included practice items that needed to be passed prior to continuing to test items. For these tasks, we calculated the percentage of children who failed practice trials as an indication of the difficulty of task demands. For WPPSI-IV tasks, because all children continued to test trials regardless of performance on sample items, we scored whether children achieved a basal level of performance.

For Zoo Locations and Picture Concepts, failure to achieve a basal level of performance was indexed by failing the first two or three test items, respectively (i.e., meeting the discontinue criteria). For Picture Memory, failure to achieve a basal level of performance was calculated separately for children younger and older than age four. For children younger than four, failure to achieve a basal level of performance was indexed by failing the first three test items. For children aged four and older, failure to achieve a basal level of performance was indexed by failing to answer items 7 and 8 correctly, and then failing to answer two consecutive items correctly during the reverse administration of items 1 through 6. For Matrix Reasoning, failure to achieve a basal level of performance was calculated separately for children younger and older than age five. For children younger than five, failure to achieve a basal level of performance was indexed by failing the first three test items. For five-year-olds, failure to achieve a basal level of performance was indexed by failing to answer items 4 and 5 correctly, and then failing to answer two consecutive items correctly during the reverse administration of items 1 through 3.

#### 2.3.3. Floor and Ceiling Effects

Children who scored at floor level failed to answer any test items correctly (i.e., received the minimum score for the task). Children who scored at ceiling level answered all test items correctly (i.e., received the maximum score for the task). Floor and ceiling rates for each task were tabulated as an additional indicator of the difficulty or ease of task demands.

### 2.4. Demographics

Parents provided demographic information about themselves and their child during the initial recruitment phone call. Child gender, race, ethnicity, and date of birth were collected, as was household income. Child age in years was subsequently calculated based on the date of birth and testing date. Child minoritized race was defined as any non-White race (i.e., African American/Black, Asian, Native American, Pacific Islander). Child minoritized ethnicity was defined as being Hispanic or Latino/a. Although three household income groups were formed for the purposes of recruitment (as described above), for the purpose of analyses, household income was dichotomized into non-low income (≥200% of the poverty threshold) and low income (<200% of the poverty threshold).

### 2.5. Analytic Plan

Data analysis proceeded in several steps. First, we compared mean accuracy scores and correlations among the three batteries. Next, we calculated and compared mean rates of failure to pass practice/achieve basal, and floor and ceiling rates for each battery. For failure to pass practice/achieve basal and floor and ceiling rates, these mean values were expressed as a proportion of tasks within each battery. Then, we conducted an exploratory factor analysis (EFA) of all tasks using percent correct scores to determine if separate factors emerged for each battery. Multiple imputations were conducted to account for missing data in the EFA. The imputation model included family and child characteristics, and children’s scores on tasks. For each missing value, a set of plausible values was estimated from the imputation model using a Bayesian E-M algorithm with bootstrapping [44] to produce 40 imputation data sets. Analyses were conducted with each data set, and results were combined taking into account variability within and between data sets. The criterion of eigenvalues larger than one and the inspection of scree plotting were used to determine the number of reliable factors [45].

We then computed percentile scores (5th through 95th) for the EF Touch battery as well as for individual tasks. We report percentiles for the entire preschool sample and separately for 3-, 4-, and 5-year-olds. Finally, we compared performance metrics among subgroups of our full sample, including children of different ages (3- vs. 4- vs. 5-year-olds), gender (male vs. female), household income level (non-low income vs. low-income), and racial (White vs. non-White) and ethnic (Hispanic vs. non-Hispanic) minoritized statuses. All analyses were conducted in SAS 9.3 (SAS Institute, Cary, NC, USA).

## 3. Results

### 3.1. Descriptive Statistics among Entire Sample

Mean accuracy scores and correlations among the three EF batteries (EF Touch, NT-ECCB, WPPSI-IV) are presented in Table 1. Overall, battery accuracy was highest for EF Touch (*M* = 0.66, *SD* = 0.15) and lowest for the WPPSI-IV (*M* = 0.36, *SD* = 0.14), with the NT-ECCB falling in the middle (*M* = 0.40, *SD* = 0.23). Comparisons among children who completed each pair of assessments indicated that children were significantly more accurate on EF Touch than on the NT-ECCB, *t*(250) = 24.81, *p* < 0.001, or on WPPSI-IV, *t*(826) = 59.59, *p* < 0.001. However, children were not significantly more accurate on the NT-ECCB compared to the WPPSI-IV, *t*(254) = 0.76, *p* = 0.45. Scores on all three EF batteries were moderately correlated with one another (*r* = 0.44–0.51, all *p* < 0.001).

Mean rates of failure to pass practice/achieve basal, and floor and ceiling effects for each battery are presented in Table 2. A higher proportion of children failed practice trials for EF Touch (*M* = 0.08, *SD* = 0.13) and the NT-ECCB (*M* = 0.08, *SD* = 0.21), compared to the WPPSI-IV (*M* = 0.04, *SD* = 0.16). Though floor effects were low for all the task batteries (range = 0.01–0.04), they were the lowest for EF Touch (*M* = 0.01, *SD* = 0.04). On the other hand, ceiling effects tended to be highest for EF Touch (*M* = 0.08, *SD* = 0.12), moderate for the NT-ECCB (*M* = 0.04, *SD* = 0.14), and non-existent for the WPPSI-IV (*M* = 0.00, *SD* = 0.00). Comparisons among children who completed each pair of assessments indicated that children failed significantly more practice trials for EF Touch, as compared to the WPPSI-IV, *t*(827) = 6.14, *p* < 0.001, but not compared to the NT-ECCB, *t*(258) = −0.52, *p* = 0.60. Children also failed more practice trials for the WPPSI-IV than for the NT-ECCB, *t*(262) = 3.16, *p* < 0.01.

We then conducted an EFA with all the tasks from all three test batteries. We anticipated three possible factor structures: (1) a single dominant factor representing general EF ability across all the batteries; (2) a three-factor structure representing the working memory, inhibitory control, and attention-shifting components of EF; or (3) a three-factor structure representing each of the task batteries. An EFA was performed to examine the dimensionality of the tasks using promax rotation. The tasks clearly loaded onto one factor, with individual loadings ranging from 0.45 to 0.71. The single factor accounted for 64% of the shared variance among the tasks. This single-factor interpretation was supported by a first eigenvalue of 4.09 and then a large decrease to the next largest eigenvalue (1.27). Two- and three-factor solutions were also estimated but cross-loading of tasks made interpretation difficult.

### 3.2. EF Touch Percentiles

Values for the 5th through 95th percentile on the overall EF Touch battery as well as individual tasks are presented in Table 3 and Table 4 respectively. Values are presented for the overall sample as well as by age. Percentile scores for EF Touch have not been reported elsewhere and are useful for understanding child performance in a comprehensive manner.

### 3.3. Descriptive Statistics among Subgroups

Next, we compared mean accuracy, failure to pass practice/achieve basal, and floor and ceiling rates among age groups (Table 5), gender (Table 6), household income level (Table 7), and racial and ethnic minoritized statuses (Table 8). We highlight notable group differences in the subsequent sections.

#### 3.3.1. Age

Mean accuracy scores on all three batteries were linearly associated with child age (all *F* > 32.68, all *p* < 0.0001), with older children demonstrating higher accuracy. The proportion of failed tasks in the EF Touch battery was inversely associated with child age, *F*(2, 827) = 12.28, *p* < 0.0001. A post hoc Tukey test showed significant differences (*p* < 0.05) between 4- and 5-year-olds, but not between 3- and 4-year-olds. Similarly, the proportion of failed tasks in the NT-ECCB was lower for 4- versus 5-year-olds, *F*(1, 261) = 14.43, *p* < 0.001. Children’s failure to achieve a basal level of performance on the WPPSI-IV tasks was not related to age, *F*(2, 840) = 0.70, *p* = 0.50.

The proportion of tasks for which children scored at floor level significantly differed by age for the EF Touch battery, *F*(2, 827) = 4.74, *p* < 0.01, with a post hoc Tukey test showing significantly fewer (*p* < 0.05) floor effects for 4- versus 3-year-olds, but no differences between 4- and 5-year-olds. For the NT-ECCB and WPPSI-IV, floor effects did not vary by age (all *F* < 3.57, all *p* > 0.06).

Finally, the proportion of tasks for which children scored at ceiling level was linearly associated with child age for the EF Touch battery, *F*(2, 827) = 10.75, *p* < 0.0001 indicating more ceiling effects for older age groups. For the NT-ECCB and WPPSI-IV, ceiling effects did not differ as a function of age (all *F* < 0.08, all *p* > 0.77).

#### 3.3.2. Gender

Few gender differences in performance were observed for any EF battery. The only significant difference in performance was on the NT-ECCB, where females (*M* = 0.12, *SD* = 0.26) failed a higher proportion of practice trials than males (*M* = 0.05, *SD* = 0.15), *t*(261) = 2.60, *p* = 0.01, *d* = 0.33.

#### 3.3.3. Household Income

There were minor differences in task performance based on household income level. For EF Touch, children from low-income households (*M* = 0.10, *SD* = 0.15) failed a higher proportion of practice trials than did children from non-low-income households (*M* = 0.07, *SD* = 0.11), *t*(828) = −3.30, *p* = 0.001, *d* = 0.23. There were no differences in task performance based on household income for the NT-ECCB. For the WPPSI-IV, children from low-income households (*M* = 0.34, *SD* = 0.14) had significantly lower accuracy scores compared to children from non-low-income households (*M* = 0.38, *SD* = 0.13), *t*(841) = 3.87, *p* < 0.001, *d* = 0.30.

#### 3.3.4. Racial/Ethnic Minority Status

For EF Touch and the WPPSI-IV, there were no significant differences in performance based on racial minority status. For the NT-ECCB, several racial differences in task performance were apparent. White children (*M* = 0.37, *SD* = 0.23) had significantly lower mean accuracy scores than non-White children (*M* = 0.44, *SD* = 0.24), *t*(254) = 2.58, *p* = 0.01, *d* = 0.30. However, White children (*M* = 0.06, *SD* = 0.16) had significantly higher rates of ceiling effects compared to non-White children (*M* = 0.02, *SD* = 0.11), *t*(254) = −1.98, *p* < 0.05, *d* = 0.29.

There were also differences in task performance based on ethnic minority status across all three batteries examined. Accuracy scores were lower for Hispanic children than for non-Hispanic children across all three batteries (all *t* > 2.41, all *p* < 0.02). Hispanic children (*M* = 0.13, *SD* = 0.17) also failed significantly more practice trials on the EF Touch battery than did non-Hispanic children (*M* = 0.07, *SD* = 0.12), *t*(828) = −5.03, *p* < 0.0001, *d* = 0.41. Finally, Hispanic (*M* = 0.06, *SD* = 0.11) children were significantly less likely to score at ceiling level on the EF Touch battery compared to non-Hispanic children (*M* = 0.08, *SD* = 0.12), *t*(828) = 2.02, *p* < 0.05, *d* = 0.17.

## 4. Discussion

The primary purpose of this study was to conduct a descriptive comparison among three performance-based EF batteries. We focused on batteries that represented different price points in the market ranging from free to significantly over $1000. In addition to comparing accuracy scores across the measures, we also sought to examine practical issues that may impact administration (i.e., failure of practice trials; floor and ceiling effects) and contrast these metrics across several demographic subpopulations.

Overwhelmingly, the batteries performed similarly across all the metrics examined. In the full sample, mean accuracy and floor and ceiling effects were comparable across the measures. Percent correct scores ranged from 36% to 66% and floor and ceiling effects (if present) were low. Children’s performance across the batteries was moderately correlated (*r* = 0.44 to 0.51). These correlations were similar to or higher than those found across other measures of EF [46]. Although this level of correlation does not suggest that the batteries can be used interchangeably, it does suggest that they have a substantial amount of overlapping variance. Second, a single common factor emerged from an EFA of all the tasks across all the batteries. This finding provides support for viewing all tasks as tapping into a single unidimensional construct of EF and indicates that the tasks within a battery do not necessarily have more in common than those across batteries.

When examining performance across the three batteries in different subpopulations, small to no differences in accuracy were found for income, gender, race, and ethnicity. Across batteries, accuracy differences were only detected for income on the WPPSI-IV and race on the NT-ECCB, with children from low-income households performing worse than children from non-low-income households and children with a minoritized race outperforming White children. It is noteworthy that across the measures, children of Hispanic ethnicity had lower scores than their non-Hispanic peers. This reduced accuracy for Hispanic populations could potentially be associated with differences in child language skills, although our data did not have information on dual-language learners (all children were assessed in English). Overall, our results suggest that the batteries performed similarly across the subpopulations we examined. Put another way, practitioners can feel confident that there is no difference between batteries in the systematic depression of scores for a particular subpopulation of interest.

Performance on all the measures was positively associated with age in this cross-sectional sample. Of particular interest, accuracy scores were considerably higher (34% to 51%) on the NT-ECCB in 5- versus 4-year-olds (scores were not available for 3-year-olds). Likewise, there was also a large drop in the percentage of children failing practice trials (12% to 1%) on the NT-ECCB in these two age groups. These results suggest that the administration of the NT-ECCB may be more successful for 5-year-old than 4-year-old children. Others have reported similar findings: Akshoomoff and colleagues [31] noted floor effects on some NT-ECCB subtests for children on the lower end of abilities, and Becker and colleagues [47] reported that 30% of 3-year-olds were unable to pass practice items required for task administration. In contrast, 5-year-olds had the greatest risk of scoring at ceiling on EF Touch, and their accuracy scores demonstrated less variability (*M* = 0.77, *SD* = 0.11) compared to other ages. It is worth noting that our 5-year-old sample was comprised of more male and minoritized children, two risk factors associated with poorer EF performance. As such, the current findings may be underestimating age-related changes in performance across batteries. Despite this, it appears that EF Touch may be more appropriate for younger preschool children, whereas the NT-ECCB may be more appropriate for older preschool children. Replication of these age-related findings is needed.

### Limitations

There are several noteworthy limitations in the current study. First, although the current sample was large and attempts were made to ensure that it was diverse, it was not a representative sample, nor did it include any clinical populations. Second, the current study only administered specific subtests from the NT-ECCB and WPPSI-IV. It is relevant to note that both batteries have additional subtests available, and there may be a clinical advantage to having information for multiple domains of cognition when factoring in the cost of an individual battery. Third, the planned missingness of the study design made NT-ECCB scores unavailable for 3-year-olds. Therefore, we were not able to make direct comparisons at age 3 across the batteries, and our results for NT-ECCB may not hold for children younger than age 4. While we provided percentile scores for the EF Touch battery, it is worth pointing out that, unlike the NT-ECCB and WPPSI-IV, norm-referenced or age-scaled scores are not available for EF Touch, and it has not been validated for use with clinical populations.

## 5. Conclusions

There are a variety of reasons a researcher may choose a particular EF assessment. In some instances, there may be a significant advantage to using a battery that provides age-referenced norm scores, and this may apply more to practitioners seeking a diagnostic instrument than to researchers. Likewise, psychometric similarities may not be meaningful if the goal is to characterize membership in a clinical population. In other instances, budgets may be limited, and there is an advantage in using a free measure. The current study aimed to provide researchers and practitioners with guidance in battery selection by comparing a free battery with two batteries available for purchase. The free EF Touch battery performed similarly across a variety of metrics when compared to the for-purchase batteries. Interestingly, although the EF Touch battery was developed using a low-income and racially diverse sample [17], it did not perform superior to the other batteries in these subpopulations. There is some evidence that the EF Touch performed better with younger children than the other batteries. For example, children had the highest accuracy scores on EF Touch, suggesting a possible advantage for using this measure in younger preschool populations. However, children also had the largest ceiling effects on EF Touch, indicating that this battery may not work as well for older or more precocious preschoolers.

It is worth noting that this research was conducted in English, although these measures are available in other languages, including Spanish. It will be important for future research to investigate if these associations hold for dual- or multi-language learners assessed in both their native and second language. Moreover, findings may also differ for populations outside of the United States, as cross-cultural issues with the measures may arise.

Practitioners have many options when selecting an EF measure and they may have concerns about how a battery will perform in their specific population of interest. To date, no research has conducted a head-to-head comparison across several of the available batteries. Practitioners may need guidance when weighing the advantages and costs associated with the use of a particular battery. Our findings suggest that the three batteries examined here are remarkably similar in performance with diverse populations and assess the same underlying construct of EF. However, practical considerations such as costs and administrative burden may be the most germane information for researchers to consider when adopting an EF assessment battery. In these comparisons, the NT-ECCB and EF Touch have an advantage with significantly less administration burden and EF Touch is the only battery available at no cost.

## Figures and Tables

**Table 1:** Mean accuracy and correlations among EF batteries.

Battery	EF Touch	NT-ECCB	WPPSI-IV
EF Touch	-		
NT-ECCB	0.50 ***	-	
WPPSI-IV	0.51 ***	0.44 ***	-
Mean	0.66	0.40	0.36
*SD*	0.15	0.23	0.14
Min	0.15	0.00	0.00
Max	0.96	0.95	0.81

Note. Sample sizes for correlations range from 251 to 827, given variation among participants’ task completion. *** *p* < 0.001.

**Table 2:** Average rates of failure to pass practice, floor effects, and ceiling effects across all EF task batteries.

Battery	*N*	Failed Practice *M* (*SD*)	Floor Effect *M* (*SD*)	Ceiling Effect *M* (*SD*)
EF Touch	830	0.08 (0.13)	0.01 (0.04)	0.08 (0.12)
NT-ECCB	256	0.08 (0.21)	0.03 (0.12)	0.04 (0.14)
WPPSI-IV	843	0.04 (0.16)	0.04 (0.16)	0.00 (0.00)

Note. *M* = mean, *SD* = standard deviation.

**Table 3:** Percentile scores for EF Touch by age.

		Percentiles						
Age Group	*N*	5th	10th	25th	50th	75th	90th	95th
3-yr-olds	296	0.34	0.38	0.46	0.58	0.67	0.76	0.81
4-yr-olds	373	0.44	0.51	0.62	0.70	0.79	0.85	0.88
5-yr-olds	161	0.58	0.63	0.72	0.80	0.85	0.89	0.91
Total sample	830	0.38	0.44	0.56	0.68	0.79	0.85	0.88

**Table 4:** Percentile scores for individual EF Tasks by age.

			Percentiles						
Task	*N*	Mean (*SD*)	5th	10th	25th	50th	75th	90th	95th
Spatial Conflict Arrows	645	0.69 (0.29)	0.12	0.18	0.35	0.65	0.82	0.94	1.00
3-yr-olds	293	0.49 (0.25)	0.12	0.18	0.29	0.47	0.71	0.82	0.88
4-yr-olds	241	0.65 (0.30)	0.06	0.24	0.41	0.71	0.88	10.00	1.00
5-yr-olds	111	0.78 (0.23)	0.29	0.35	0.71	0.82	0.94	10.00	1.00
Silly Sounds Stroop	591	0.58 (0.27)	0.12	0.24	0.35	0.59	0.82	0.84	1.00
3-yr-olds	245	0.44 (0.24)	0.12	0.18	0.29	0.41	0.59	0.76	0.94
4-yr-olds	238	0.64 (0.26)	0.18	0.24	0.41	0.71	0.88	0.94	1.00
5-yr-olds	108	0.76 (21)	0.35	0.47	0.65	0.82	.94	1.00	1.00
Animal Go/No-Go	563	0.81 (0.24)	0.25	0.50	0.75	0.88	1.00	1.00	1.00
3-yr-olds	233	0.72 (0.29)	0.13	0.25	0.63	0.88	1.00	1.00	1.00
4-yr-olds	230	0.86 (0.18)	0.50	0.63	0.75	0.88	1.00	1.00	1.00
5-yr-olds	100	0.89 (0.13)	0.63	0.75	0.88	0.88	1.00	1.00	1.00
Working Memory Span	475	0.56 (0.20)	0.22	0.33	0.44	0.56	0.72	0.83	0.83
3-yr-olds	131	0.50 (0.24)	0.08	0.17	0.33	0.50	0.72	0.83	0.83
4-yr-olds	231	0.54 (0.17)	0.28	0.33	0.44	0.56	0.67	0.78	0.78
5-yr-olds	113	0.65 (0.17)	0.33	0.39	0.56	0.67	0.78	0.89	0.89
Pick the Picture	467	0.70 (0.13)	0.47	0.53	0.63	0.72	0.78	0.84	0.88
3-yr-olds	121	0.68 (0.14)	0.42	0.50	0.59	0.67	0.75	0.83	0.86
4-yr-olds	243	0.70 (0.12)	0.50	0.53	0.63	0.72	0.78	0.81	0.84
5-yr-olds	103	0.75 (0.14)	0.53	0.63	0.69	0.75	0.84	0.88	0.91
Something’s the Same	807	0.73 (0.14)	0.50	0.53	0.63	0.73	0.83	0.90	0.93
3-yr-olds	282	0.66 (0.13)	0.47	0.50	0.57	0.67	0.73	0.80	0.87
4-yr-olds	368	0.75 (0.13)	0.53	0.57	0.67	0.77	0.87	0.90	0.93
5-yr-olds	157	0.82 (0.11)	0.60	0.67	0.73	0.83	0.90	0.93	0.97

**Table 5:** Descriptive statistics for all EF batteries by age group.

Battery	*N*			Accuracy *M* (*SD*)			Failed Practice *M* (*SD*)			Floor Effect *M* (*SD*)			Ceiling Effect *M* (*SD*)		
	3	4	5	3	4	5	3	4	5	3	4	5	3	4	5
EF Touch	284	375	171	0.57 ^a^ (0.15)	0.69 ^b^ (0.13)	0.77 ^c^ (0.11)	0.09 ^a^ (0.14)	0.09 ^a^ (0.14)	0.04 ^b^ (0.08)	0.01 ^a^ (0.05)	0.00 ^b^ (0.03)	0.00 ^b^ (0.03)	0.05 ^a^ (0.10)	0.08 ^b^ (0.12)	0.11 ^c^ (0.12)
NT-ECCB	0	170	86		0.34 ^a^ (0.22)	0.51 ^b^ (0.23)		0.12 ^a^ (0.25)	0.01 ^b^ (0.08)		0.04 ^a^ (0.14)	0.01 ^a^ (0.05)		0.04 ^a^ (0.14)	0.05 ^a^ (0.15)
WPPSI-IV	287	384	172	0.31 ^a^ (0.12)	0.36 ^b^ (0.14)	0.44 ^c^ (0.14)	0.05 ^a^ (0.20)	0.04 ^a^ (0.15)	0.03 ^a^ (0.13)	0.05 ^a^ (0.20)	0.04 ^a^ (0.15)	0.03 ^a^ (0.13)	0.00 ^a^ (0.00)	0.00 ^a^ (0.00)	0.00 ^a^ (0.00)

Note. Values within a row with different superscripts are significantly different (*p* < 0.05). *M* = mean, *SD* = standard deviation.

**Table 6:** Descriptive statistics for all EF batteries by gender.

Battery	Accuracy M (SD)		Failed Practice		Floor Effect		Ceiling Effect	
	Female	Male	Female	Male	Female	Male	Female	Male
EF Touch	0.67 (0.15)	0.66 (0.15)	0.08 (0.13)	0.08 (0.14)	0.01 (0.04)	0.01 (0.04)	0.08 (0.12)	0.07 (0.12)
NT-ECCB	0.42 (0.22)	0.38 (0.24)	**0.12** **(0.26)**	0.05 * **(0.15)**	0.03 (0.02)	0.02 (0.12)	0.04 (0.14)	0.04 (0.14)
WPPSI-IV	0.37 (0.14)	0.35 (0.14)	0.03 (0.15)	0.04 (0.17)	0.03 (0.15)	0.04 (0.17)	0.00 (0.00)	0.00 (0.00)

Note. Adjacent values that are bolded are significantly different from one another. * *p* < 0.05. *M* = mean, *SD* = standard deviation.

**Table 7:** Descriptive statistics for all EF batteries by household income.

Battery	Accuracy *M* (*SD*)		Failed Practice		Floor Effect		Ceiling Effect	
	Non-Low Income	Low-Income	Non-Low Income	Low-Income	Non-Low Income	Low-Income	Non-Low Income	Low-Income
EF Touch	0.67 (0.15)	0.65 (0.16)	**0.07** **(0.11)**	0.10 ** **(0.15)**	0.01 (0.04)	0.01 (0.04)	0.08 (0.12)	0.07 (0.11)
NT-ECCB	0.41 (0.23)	0.39 (0.24)	0.06 (0.20)	0.10 (0.23)	0.02 (0.12)	0.03 (0.12)	0.05 (0.15)	0.03 (0.12)
WPPSI-IV	**0.38** **(0.13)**	0.34 *** **(0.14)**	0.03 (0.14)	0.05 (0.19)	0.03 (0.14)	0.05 (0.19)	0.00 (0.00)	0.00 (0.00)

Note. Adjacent values that are bolded are significantly different from one another. ** *p* < 0.01. *** *p* < 0.001. *M* = mean, *SD* = standard deviation. Households were categorized as low-income if total income was <200% US federal poverty threshold for a given household size.

**Table 8:** Descriptive statistics for all EF batteries by child minority race/ethnicity status.

Battery	Accuracy *M* (*SD*)				Failed Practice				Floor Effect				Ceiling Effect			
	White	Non-White	Non-Hispanic	Hispanic	White	Non-White	Non-Hispanic	Hispanic	White	Non-White	Non-Hispanic	Hispanic	White	Non-White	Non-Hispanic	Hispanic
EF Touch	0.66 (0.15)	0.67 (0.16)	**0.68** **(0.15)**	0.61 *** **(0.15)**	0.08 (0.13)	0.09 (0.14)	**0.07** **(0.12)**	0.13 *** **(0.17)**	0.01 (0.04)	0.00 (0.03)	0.01 (0.04)	0.01 (0.05)	0.08 (0.11)	0.07 (0.12)	**0.08** **(0.12)**	0.06 * **(0.11)**
NT-ECCB	**0.37** **(0.23)**	0.44 * **(0.24)**	**0.42** **(0.23)**	0.33 * **(0.22)**	0.08 (0.22)	0.09 (0.21)	0.08 (0.22)	0.11 (0.21)	0.03 (0.14)	0.01 (0.08)	0.02 (0.11)	0.05 (0.16)	**0.06** **(0.16)**	0.02 * **(0.11)**	0.05 (0.14)	0.03 (0.12)
WPPSI-IV	0.36 (0.13)	0.36 (0.15)	**0.37** **(0.14)**	0.32 *** **(0.13)**	0.03 (0.15)	0.05 (0.19)	0.04 (0.16)	0.05 (0.17)	0.03 (0.15)	0.04 (0.18)	0.04 (0.16)	0.04 (0.17)	0.00 (0.00)	0.00 (0.00)	0.00 (0.00)	0.00 (0.00)

Note. Adjacent values that are bolded are significantly different from one another. * *p* < 0.05. *** *p* < 0.001. *M* = mean, *SD* = standard deviation.

## Data Availability

The data presented in this study are available on request from the corresponding author due to privacy concerns.
